# Characterisation of the legume *SERK-NIK *gene superfamily including splice variants: Implications for development and defence

**DOI:** 10.1186/1471-2229-11-44

**Published:** 2011-03-09

**Authors:** Kim E Nolan, Sergey Kurdyukov, Ray J Rose

**Affiliations:** 1 Australian Research Council Centre of Excellence for Integrative Legume Research, School of Environmental and Life Sciences. The University of Newcastle. University Dr. Callaghan, NSW, 2308, Australia

## Abstract

**Background:**

*SOMATIC EMBRYOGENESIS RECEPTOR-LIKE KINASE *(*SERK*) genes are part of the regulation of diverse signalling events in plants. Current evidence shows SERK proteins function both in developmental and defence signalling pathways, which occur in response to both peptide and steroid ligands. *SERKs *are generally present as small gene families in plants, with five *SERK *genes in Arabidopsis. Knowledge gained primarily through work on Arabidopsis SERKs indicates that these proteins probably interact with a wide range of other receptor kinases and form a fundamental part of many essential signalling pathways. The *SERK1 *gene of the model legume, *Medicago truncatula *functions in somatic and zygotic embryogenesis, and during many phases of plant development, including nodule and lateral root formation. However, other *SERK *genes in *M. truncatula *and other legumes are largely unidentified and their functions unknown.

**Results:**

To aid the understanding of signalling pathways in *M. truncatula*, we have identified and annotated the *SERK *genes in this species. Using degenerate PCR and database mining, eight more *SERK*-like genes have been identified and these have been shown to be expressed. The amplification and sequencing of several different PCR products from one of these genes is consistent with the presence of splice variants. Four of the eight additional genes identified are upregulated in cultured leaf tissue grown on embryogenic medium. The sequence information obtained from *M. truncatula *was used to identify *SERK *family genes in the recently sequenced soybean (*Glycine max*) genome.

**Conclusions:**

A total of nine *SERK *or *SERK-like *genes have been identified in *M. truncatula *and potentially 17 in soybean. Five *M. truncatula SERK *genes arose from duplication events not evident in soybean and Lotus. The presence of splice variants has not been previously reported in a *SERK *gene. Upregulation of four newly identified *SERK *genes (in addition to the previously described *MtSERK1*) in embryogenic tissue cultures suggests these genes also play a role in the process of somatic embryogenesis. The phylogenetic relationship of members of the *SERK *gene family to closely related genes, and to development and defence function is discussed.

## Background

The plant receptor-like kinases (RLKs) are a large group of signalling proteins in plants, and are a fundamental part of plant signal transduction. In Arabidopsis the RLK family contains more than 600 members, constituting 60% of kinases, including almost all of the transmembrane kinases [1]. The position of RLKs in the plasma membrane, with an extracellular receptor domain and an intracellular kinase domain, makes them well suited to the task of perceiving a signal external to the cell and conducting that signal into the cell in order to elicit a response. In addition to RLKs there are a number of receptor-like proteins (RLPs). These proteins contain an extracellular domain similar to a RLK but lack the intracellular kinase domain [2]. Based on the criteria of extracellular domain structure and kinase domain phylogeny, RLKs are divided into subfamilies [1]. The *SOMATIC EMBRYOGENESIS RECEPTOR-LIKE KINASE *(*SERK) *gene family belong to the leucine-rich repeat (LRR) subfamily of RLKs. These RLKs contain varying numbers of LRRs in their extracellular receptor domain. *SERK *genes belong to subgroup II (LRRII) and contain five LRR domains [1].

The family has been defined according to several factors. The first is the presence of 11 exons with conserved splicing boundaries and the tendency for each exon to encode a specific protein domain. Secondly the SERK amino acid sequence contains a particular order of domains from N to C-terminal: Signal peptide (SP), leucine zipper (ZIP), 5 LRRs, a proline-rich domain (SPP), transmembrane, kinase and C-terminal domains. The SPP domain, containing the SPP motif and the C-terminal domain are considered to be the characteristic domains of SERK proteins [3,4]. Although this is largely correct for annotated *SERK *genes there is some divergence from the set criteria. The Arabidopsis *NIK *(*NSP interacting kinase*) genes share many similarities with *SERK *genes. *NIK *genes are so named because of their function in signalling during virus infection [5,6]. They are described as interacting with the Nuclear Shuttle Protein (NSP) domain of the virus.

The first *SERK *genes identified were linked to competence of cultured cells to form somatic embryos in carrot (*Daucus carota*), orchard grass (*Dactylis glomerata*) and *Arabidopsis thaliana *species [3,7,8]. Since that time *SERK *gene expression has been associated with somatic embryogenesis (SE) and organogenesis in numerous species [9-19]. In Arabidopsis five *SERK *genes have been identified [3] (*AtSERKs 1-5*) and the gene functioning in SE is *AtSERK1 *(locus At1g71830). As understanding of the roles of the different members of the *SERK *gene family has increased, it has become apparent that these genes function in diverse signalling pathways with roles from development to defence. The Arabidopsis *SERK *gene family is subdivided into two subfamilies, generated from an ancestral gene duplication event. The first subfamily consists of *AtSERKs 1 *and *2 *(SERK1/2) and the second subfamily, *AtSERKs 3*, *4 *and *5 *(SERK3/4/5) [3,20,21].

AtSERK1 is required in conjunction with AtSERK2 for anther development and male gametophyte maturation, with double mutants lacking a tapetal layer and failing to develop pollen [22,23]. AtSERK1 and AtSERK3 (also called BRI1-associated kinase1 (BAK1)) function in brassinosteroid (BR) signal transduction as components of the BR receptor complex, through dimerization with brassinosteroid-insensitive 1 (BRI1) kinase [24-26]. Both AtSERK3 and AtSERK4 (also called BAK1-LIKE 1 (BKK1)) have been linked to programmed cell death, which can function in both developmental and pathogen defence roles [20,27]. What has emerged from studies of Arabidopsis SERK signalling is that these genes have a tendency to be redundant in pairs with different pairs working in different pathways. Therefore single *SERK *gene mutants show weak or no phenotype as a second *SERK *gene can complement their function. Different combinations of *SERK *genes act in different pathways and these combinations vary according to the pathway. For instance, AtSERK1 and 2 can complement each other in anther development, where AtSERK3 is shown not to function [21]. However, AtSERK1 and 3 function together in BR signalling, and AtSERK3 and 4 are redundant in the programmed cell death pathway. So far a function for AtSERK5 is not known.

In defence responses, AtSERK3/BAK1 functions in pathogen-associated molecular pattern (PAMP)-triggered immunity through heterodimerization with the Flagellin sensing 2 (FLS2) receptor kinase in response to binding by the bacterial PAMP, flagellin [28,29]. A rice SERK, OsSERK1, shows activity in both somatic embryogenesis and fungal defence [30]. The concept of a receptor functioning in both development and pathogen response pathways is reminiscent of the TOLL receptor of Drosophila, also an LRR protein, which is a controlling factor in both embryo development and immunity [28]. Similarly ERECTA in Arabidopsis functions in inflorescence and fruit development as well as pathogen resistance [31].

The ability of AtSERKs to be essential to a number of diverse pathways, receptive to both peptide and steroid ligands, poses the question as to how these similar proteins can show such diversity of function. One possibility is that they are not the primary ligand-binding receptor protein, but instead dimerize with other RLK proteins that are specifically targeted to the one response pathway; for example, the BRI1 RLK in the case of BR signalling, or the FLS2 RLK in immune response to bacterial infection [32]. There is also evidence that AtSERK proteins may function in the process of endocytosis of the active receptor complex following ligand binding [28,33,34].

In the model legume *M. truncatula *we have studied *MtSERK1 *in relation to SE and other aspects of development [9,35] but no additional information is available in legumes on other members of the *SERK *family. Legume species comprise some of the world's essential crops for both human and animal nutrition, as a source of biofuels and are of ecological importance due to their ability to form symbiotic relationships with *Rhizobium *species and fix atmospheric nitrogen [36]. In this study we have identified members of the *SERK *family in *M. truncatula *and soybean (*Glycine max*) and analysed their phylogeny in relation to development and defence. In the case of *MtSERK3 *a number of transcripts have been identified by PCR, consistent with the presence of splice variants, and this is discussed in relation to MtSERK3 function.

## Results

### *SERK *genes identified in *M. truncatula*

Using degenerate PCR from various tissues and database mining we identified eight putative *SERK *genes in *M. truncatula*, in addition to the already characterized *MtSERK1 *(Table 1). Degenerate PCR did not detect any *SERK*-like sequences that were not also found using database searches. Based on our analysis these genes were named *MtSERK 2-6 *and *MtSERK-like 1-3 *(*MtSERKL 1-3*). Five of the genes had one or two corresponding tentative consensus (TC) or EST sequences on the DFCI Medicago gene index (http://compbio.dfci.harvard.edu/tgi/cgi-bin/tgi/gimain.pl?gudb=medicago; shown in Table 1) but none of these represented full length coding sequences. The remaining three genes (*MtSERK3, MtSERK4 and MtSERK6*) matched genomic DNA sequences but had no corresponding ESTs. Of the eight predicted genes, five (*MtSERKs 2-6*) occur in tandem over a 33 Kb region on chromosome 2 (genomic sequence from GenBank accession numbers AC195567 and AC187356). The other three occur on chromosomes 3, 5 and 8 (genomic sequences from GenBank accession numbers CT967306, CT025841 and AC126784 repectively; Table 1). PCR amplification of cDNA from various tissues and sequencing were used to obtain the full length coding sequence of each of the eight identified genes. For one of these genes, seven different cDNA sequences were amplified using nested PCR and sequenced. The presence of these different sequences is consistent with the presence of splice variants. Blastp searches of all of the predicted amino acid sequences of the putative *SERK *genes on the NCBI database http://www.ncbi.nlm.nih.gov showed *MtSERKs 2-6 *have high similarity to *AtSERK3*. The other three *MtSERKL *genes are similar to *SERKs *from various species, but in Arabidopsis, *MtSERKL1 *and *MtSERKL2 *are more similar to *NIK *genes. The homology of the *M. truncatula *SERK and SERKL sequences with each other and with Arabidopsis SERK and NIK sequences is shown in Additional file 1.

**Table 1:** *SERK *and *SERKL *genes identified in *M. truncatula*

Gene name	Genomic identifier	Chr	TC/EST identified	Current TC number	No of ESTs on DFCI	Deg PCR	Matching probeset ID on MtGI	Chr Pos (Kbp)	Gene loci (Medtr-)	SV	GenBank Number	Protein length	MW	pI
MtSERK1	AY162177	0		TC142011	10	yes	Mtr.43625.1.S1_at				AY162176	627	69140.3	5.48

MtSERK2	AC195567 AC187356	2	TC100619 TC97176	TC150247	5	yes	Mtr.37421.1.S1_at	1603.3-1609.6	2g008470 2g008480		HM640001	619	68538.8	5.47

MtSERK3	AC195567 AC187356	2			0	no	none present	1610.0 - 1616.1	2g008490 2g008500	SV1	HM640008	586	65127.2	5.12
										
										SV2	HM769882	271	29246.0	4.98
										
										SV3	HM769883	562	62537.2	5.20
										
										SV4	HM769884	247	26656.1	5.22
										
										SV5	HM769885	154	16964.2	4.59
										
										SV6	HM769886	154	16964.2	4.59
										
										SV7	HM769887	154	16964.2	4.59
										
MtSERK4	AC195567 AC187356	2			0	no	none present	1615.7-1621.4	2g008510		HM640002	615	67882.3	5.50

MtSERK5	AC195567	2	TC104947 TC110830	TC155497 TC151948	8	yes	Mtr.39468.1.S1_at Mtr.11713.1.S1_at	1622.7-1628.9	2g008520		HM640003	620	68615.9	5.61

MtSERK6	AC195567	2			0	no	none present	1629.2-1636.2	2g008530 2g008540		HM640004	642	70720.3	5.41

MtSERKL1	AC126784	8	CB891120	TC143055	4	no	Mtr.15874.1.S1_s_at Mtr.15874.1.S1_at	35000.0-35005.0	8g144660		HM640005	640	70293.2	6.66

MtSERKL2	CT025841	5	TC109616	TC150718	5	no	Mtr.41552.1.S1_at	14476.6-14481.4	5g035120		HM640006	625	69142.3	6.86

MtSERKL3	CT967306	3	TC97017	TC166655	10	no	Mtr.44258.1.S1_at	24728.8-24736.2	3g101870		HM640007	609	68019.8	5.64

Summary of *SERK *and *SERKL *genes in *M. truncatula *including splice variants (SV1-7) of *MtSERK3*. Gene name refers to the final name given to each gene. The genomic identifier is the GenBank number of the genomic sequence containing each gene. Chr is chromosome number. TC/EST identified refers to any matching TC or EST sequence found on the DFCI Medicago gene index at the time the eight new genes were first identified. These numbers have since been updated and sometimes divided into separate sequences. Current TC number shows the current corresponding TC numbers for each sequence. No. of ESTs on DFCI is the number of ESTs used to compile each TC sequence on the DFCI Medicago gene index. Detected on degenerate PCR indicates which sequences we found using that technique. Matching probeset ID on MtGI indicates the corresponding probeset on the *M. truncatula *Gene Expression Atlas. Chr Pos is the gene position in kilobase pairs (Kbp) on each chromosome established from CViT blast searches. Gene loci indicates the gene locus number/s present at each site. Splice variant (SV) numbers of the 7 MtSERK3 SVs are given. GenBank numbers apply to full-length mRNA sequences deposited on the NCBI database. Length, molecular weight (MW) and pI values of predicted protein sequences are shown.

In order to determine the chromosomal position of each gene genomic full-length coding sequences plus several hundred bases 5' and 3' of each gene were used for a CViT blast search of the *M. truncatula *pseudomolecule: MT3.0 database. Each of the Medicago *SERK *and *SERKL *genes, except for *MtSERK1*, showed 100% match to the database, and the position of these is shown in Table 1. *MtSERK1 *is not present on this database, with its closest match corresponding to part of *MtSERK2 *sequence on chromosome 2. The gene loci numbers are also shown in Table 1, with *MtSERKs2*, *3 *and *5 *each occupying two loci.

### Predicted motifs in Medicago genes and comparison with Arabidopsis SERKs

**Figure 1:**
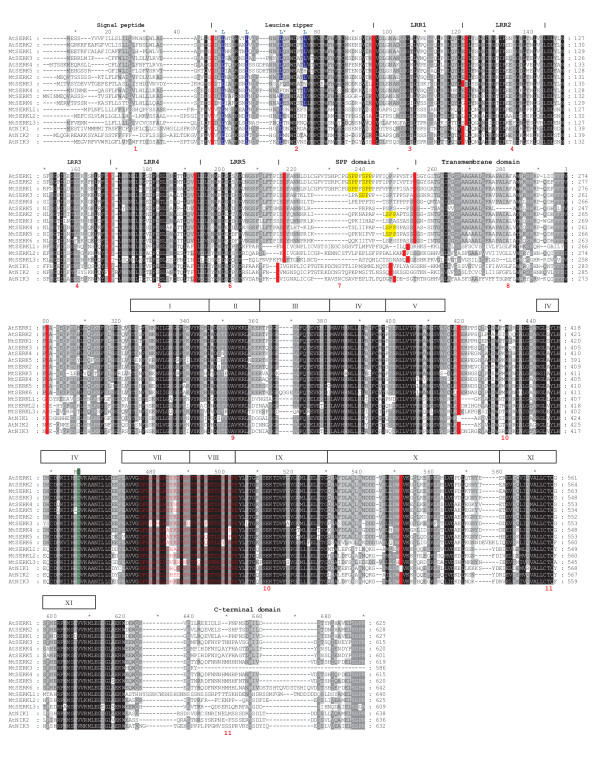
**Alignment of all 5 Arabidopsis SERKs, three Arabidopsis NIKs and *M. truncatula *SERK and SERK-like amino acid sequences**. The positions of exon boundaries are shown on each sequence with a red vertical line. Exon numbers are shown in red text below the sequence alignment. Positions of SERK protein domains are shown above the alignment. Boxed areas with Roman numerals indicate the 10 subdomains of the kinase domain. Conserved leucines of the leucine zipper are highlighted blue. The SPP motif of the SPP domain is highlighted yellow. The conserved catalytic aspartate residue in subdomain VI of the kinase domain is highlighted green and the conserved arginine of RD protein kinases immediately preceding the conserved asparatate is indicated with an R above the alignment [68]. The activation loop in subdomains VII and VII is shown in red text.

**Figure 2:**
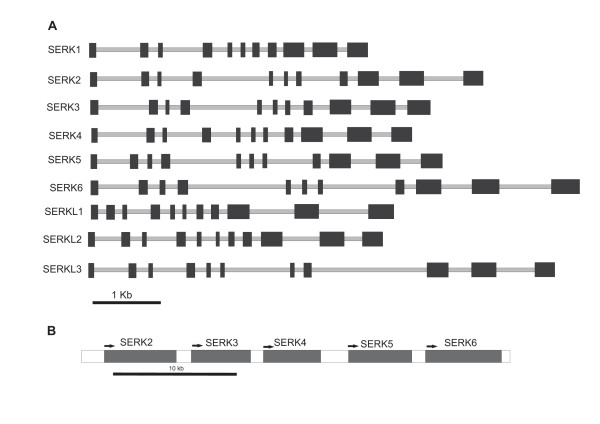
**Genomic structure of MtSERK1 and each SERK or SERKL gene obtained from genomic information on the NCBI database and from cDNA sequencing.** A.  Exons are shown as dark boxes and introns in light grey. Gene sizes are shown from the start to the stop codon. Each gene contains 11 exons. B. The relative position and size of the coding regions of the five SERK genes on chromosome 2. Arrows indicate the direction of transcription.

The positions of the different SERK domains in Arabidopsis SERKs are indicated above the sequence alignment in Figure 1. All of the *M. truncatula *sequences except for MtSERK3 have a predicted signal peptide. MtSERK3 is predicted to be secreted in a non-classical manner. The consensus sequence of a leucine zipper Leu-X_6_-Leu-X_6_-Leu-X_6_-Leu, where X is any residue [37] is present in MtSERKs 1, 2, 5 and 6. It is absent in the remaining *M. truncatula *SERK-like proteins and is also absent in Arabidopsis SERKs 4 and 5 as well as the three Arabidopsis NIKs. All of these proteins have partial leucine zipper sequences, with the first Leu-X_6_-Leu sequence intact, but lack other conserved leucines and/or have extra residues between conserved leucines (Figure 1). The positions of the five SERK LRRs are indicated in Figure 1. There is good alignment of the LRRs with the exception of LRR 5 in the three Medicago SERKL proteins. The SPP domain is not well conserved. The SERK-characteristic SPP motif, highlighted yellow in Figure 1 is not present in all SERK proteins with AtSERKs 4 and 5 lacking this motif. In *M. truncatula *the SPP motif is present in MtSERKs 1, 2, 4 and 5, but is lacking in the other proteins. The Medicago SERKL proteins show the least amount of homology in this domain. All of the *M. truncatula *sequences contain predicted transmembrane and kinase domains. The genomic structure of each of the *M. truncatula SERK *and *SERKL *genes and the relative positions of the *SERK *genes on chromosome 2 are shown in Figure 2. Each of the genes contains 11 exons which is characteristic of *SERK *genes. The gene encoding several putative splice variants is *MtSERK3*. One of the splice variants contains the usual *SERK *exon structure with eleven exons as shown in Figure 2. The main variation in the gene structure between the different *M. truncatula *genes is in the length of the introns.

Another characteristic of *SERK *genes is conservation of exon boundary sites with the tendency for different protein domains to be encoded by separate exons [4]. The positions of each exon boundary site in each sequence are shown in Figure 1. Each of the *M. truncatula *sequences identified and the Arabidopsis NIKs have similar boundary sites to the Arabidopsis SERKs, with the exception of AtNIK1, which is missing two boundary sites, with a single exon encoding the equivalent of exons 9, 10 and 11 in the other genes. The boundaries of greatest divergence occur between exons 6/7 and 7/8. Exons 6, 7 and 8 encode LRR5, the SPP and the transmembrane domains respectively.

### *SERK *gene prediction from the soybean genome

Soybean (*Glycine max*) has three genes annotated as *SERK *genes on the NCBI database. However two of these sequences (GenBank numbers EU869193 and FJ014794) are sequences from the same gene. The other sequence is Genbank number EU888313. There is also one annotated *NIK *gene in soybean (GenBank number FJ014718). To identify other putative *SERK *and *SERK*-like genes in soybean, the mRNA sequences of the *M. truncatula SERK *and *SERK*-like genes were blasted against the genomic sequence of soybean. Fourteen more *SERK-like *genomic sequences were obtained, and from these mRNA and amino acid sequences were predicted.

### Phylogenetic analysis of legume *SERK *genes

**Figure 3:**
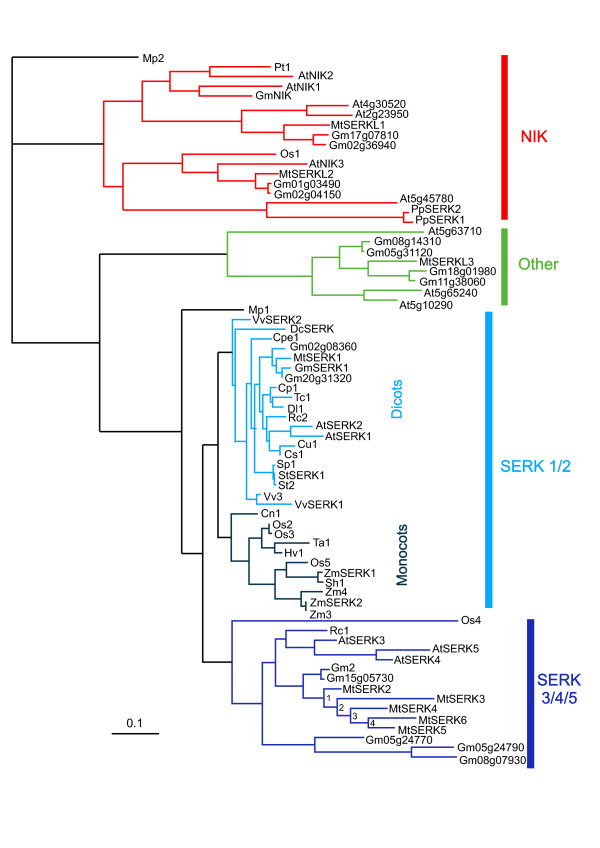
**Phlyogenetic analysis of protein sequences from all Arabidopsis RLK-LRR subclass LRRII genes, Medicago SERK and SERKL genes, known and predicted NIK and SERK-like protein sequences from soybean and SERK or SERK-like genes from a number of different species**. The soybean sequences that were predicted from genomic sequence are indicated by their gene locus number preceded by "Gm." The loci numbers of soybean protein sequences from the protein database are Gm10g36280 (GmSERK1), Gm08g19270 (Gm2) and Gm13g07060 (GmNIK). Sequences falling into the SERK1/2 subfamily are indicated with blue lines-sequences from dicotyledonous plants in light blue and from monocotyledonous plants in dark blue. The SERK3/4/5 subfamily is indicated with purple lines. Other non-SERK, non-NIK genes are a sister clade to these (shown in green). Sequences belonging to the NIK family clade are indicated with red lines. Sequences from the primitive Bryophyte, *Marchantia polymorpha*, Mp1 and Mp2, sit separately from the other family genes, but could be classed as a SERK and a NIK gene respectively. Estimated times of duplication events (indicated by numbers 1-4) in *M. truncatula *SERK 3/4/5 subfamily genes are: 1 - 3.25, 2 - 3.05, 3 - 2.65 and 4 - 2.2 million years ago. Plant species abbreviations used in tree. At - *Arabidopsis thaliana*, Cp - *Carica papaya *(papaya), Cs - *Citris sinensis *(sweet orange), Cu - *Citrus unshiu *(Satsuma orange), Cn - *Cocus nucifera *(coconut), Cpe - *Cyclamen persicum*, Dc - *Daucus carota *(carrot), Dl - *Dimocarpus longan *(logan), Gm - *Glycine max *(soybean), Hv - *Hordeum vulgare *(barley), Mp - *Marchantia polymorpha *(liverwort), Mt - *Medicago truncatula *(barrel medic), Os - *Oryza sativa *(rice), Pp - *Poa pratensis *(Kentucky bluegrass), Pt - *Populus tomentose *(Chinese white poplar), Rc - *Ricinus communis *(castor oil plant), Sh - *Saccharum hybrid cultivar *(sugarcane), *Solanum peruvianum *(Peruvian nightshade), St - *Solanum tuberosum *(potato), Tc - *Theobroma cacao *(cocoa), Ta - *Triticum aestivum *(bread wheat), Vv - *Vitis Vinifera *(grape), Zm - *Zea mays *(maize). Locus number or sequence identifier for the sequences shown are: AtSERK1 - At1G71830, AtSERK2 - At1G34210, AtSERK3 - At4G33430, AtSERK4 - At2g13790, AtSERK5 - At2G13800, AtNIK1 - At5g16000, AtNIK2 - At3g25560, AtNIK3 - At1G60800, Cp1 - ABS32233.1, Cs1 - ACP20180.1, Cu1 - BAD32780.1, Cn1 - AAV58833.2, Cpe1 - ABS11235, DcSERK - AAB61708.1, Dl1 - ACH87659.2, GmSERK1 - ACJ64717.1, Gm2 - ACJ37402.1, GmNIK - ACM89473.1, Hv1 - ABN05373.1, Mp1 - BAF79935.1, Mp2 - BAF79962.1, MtSERK1 - AAN64293.1, other *M. truncatula *genes - see Table 1, Os1 - Os01g0171000, Os2 - Os08g0174700, Os3 - Os08g07760, Os4 - Os06g0225300, Os5 - Os04g0457800, PpSERK1 - CAH56437.1, PpSERK2 - CAH56436.1, Pt1 - ABG73621.1, Rc1 - XP_002520361.1, Rc2 - XP_002534492.1, Sh1 - ACT22809.1, Sp1 - ABR18800.1, StSERK1 - ABO14173.1, St2 - ABO14172.1, Tc1 - AAU03482.1, Ta1 - ACD49737.1, VvSERK1 - CAO64642.1, VvSERK2 - CAN65708.1, Vv3 - XP_002270847.1, ZmSERK1 - NP_001105132.1, ZmSERK2 - NP_001105133.1, Zm3 - ACL53442.1, Zm4 - ACF87700.1 Other Arabidopsis RLK-LRRII sequences are labelled with their gene locus number. Associated publications: Cu1 (CitSERK1 [12], Cn1 [17], DcSERK [7], Mp1 (MpRLK2) and Mp2 (MpRLK29 [40], MtSERK1 [9], Os2 (OsSERK1 [69,70], Os3 (OsBISERK1 [43], Os4 (OsSERK3 [70], Os5 (OsSERK1 [30] and OsSERK2 [70], PpSERK1, PpSERK2 [44], StSERK1 [15], Tc1 [71], VvSERK1 and VvSERK2 [14], ZmSERK1 and ZmSERK2 [4]

A phylogenetic tree was constructed from the predicted amino acid sequences of the *M.truncatula SERK *and *SERK*-like genes, the three soybean *SERK *and *NIK *genes present in the database and the fourteen soybean genes predicted from the soybean genome sequence. Also included in the tree are all LRRII subgroup RLK-LRR genes from Arabidopsis and SERKs from the NCBI database representing full length AA sequences from a number of other plant species (Figure 3). As indicated by the blast searches some of the *M. truncatula *sequences form a clade with the known SERKs. MtSERKL1 and MtSERKL2 fall into a clade with the soybean and Arabidopsis NIKs. Sequences of four of the predicted soybean genes also fall in the NIK clade. One Medicago sequence, MtSERKL3, along with three Arabidopsis sequences and four of the predicted soybean sequences form a clade that is separate from the SERK and NIK clades (Labelled "Other" in Figure 3). The four non-Arabidopsis, non-legume sequences that fall in the NIK clade (Pt1, Os1, PpSERK1 and PpSERK2 in Figure 3) have been annotated as SERKs in the literature and/or on the NCBI database. This phylogenetic analysis shows that the five sequences from chromosome 2 that have been named as MtSERK2-6 are part of the SERK3/4/5 family clade, with MtSERK1 the only *M. truncatula *sequence in the SERK1/2 subfamily. One known and two predicted soybean sequences fall into the SERK1/2 subfamily. One known and four predicted soybean sequences fall into the SERK3/4/5 subfamily. Together the phylogenetic and exon boundary results indicate high similarity between the *SERK *and *NIK *genes. The *M. truncatula *sequences have been deposited on the NCBI database (For GenBank numbers see Table 1).

In the SERK3/4/5 subfamily, two soybean genes lie adjacent on chromosome 5, (Glyma05g24770 and Glyma05g24790) but there is not a region with five genes in tandem as is found on chromosome 2 in *M. truncatula*. *Lotus japonicus *is more closely related to *M*. *truncatula *than soybean [38]. A search of the database revealed only one Lotus predicted gene similar to the Medicago SERK3/4/5 genes. This gene occurs on chromosome 6 (Genbank accession number AP006424), which is syntenic to *M. truncatula *chromosome 2 [39]. This Lotus genomic DNA sequence showed sequence homology with all five Medicago SERK3/4/5 genes, with some sequence homology in introns and in 5' and 3' untranslated regions, as well as in exons. These results, combined with the fact that no other potential sequences were found in the Lotus genome, indicate that the single *SERK *gene region on Lotus chromosome 6 probably corresponds to the five *SERK *gene region on *M. truncatula *chromosome 2. These five *SERK *genes in Medicago may have duplicated since it diverged from Lotus. At this point it is unknown whether legumes closely related to Medicago also have replication of this *SERK *gene as there is as yet no sequence information. The intron sequences of the five replicated *M. truncatula *genes were used to estimate the times of duplication of these genes. It is estimated that duplication events occurred at 3.25, 3.05, 2.65 and 2.2 million years ago as indicated in Figure 3.

### *MtSERK3 *transcripts

**Figure 4:**
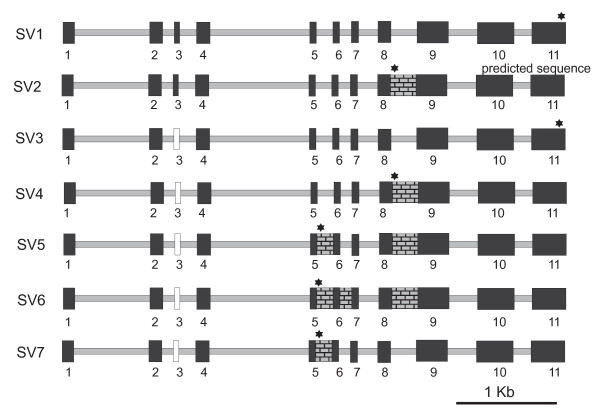
**Representation of the seven splice variants (SVs) identified from the *MtSERK3 *gene**. The exons which comprise the regular *SERK *gene structure are shown as wide dark rectangles (numbered) on a thin grey line representing introns. SV1 contains eleven exons with the structure of a typical *SERK *gene. The other splice variants have one or a combination of retained intron sequences and/or loss of exon 3 in the mRNA transcript. In transcripts missing exon 3 this exon is shown as a white rectangle. Included introns are shown as grey hatched areas. The star above each sequence is in the position of the predicted stop codon. SVs 5, 6 and 7 all encode the same amino acid sequence although their transcripts differ 3' of the stop codon. SV4 was only sequenced up to exon 10 position so it is possible there was some more variation in the region of the last two exons.

PCR analysis suggested a total of seven different transcripts consistent with seven splice variants of *MtSERK3 *. The differences observed between the splice variants is that they either include an intron or introns in their sequence and/or are missing exon 3 (Figure 4). Introns that are included as exons are introns 5, 6 and 8, either alone or in combination. Each of these intron sequences introduces a stop codon thereby creating a truncated coding sequence. Splice variant (SV) 1 has the structure of a normal *SERK *gene, containing 11 exons. SV3 is also full length except it lacks exon 3, which encodes the first LRR. SV2 and SV4 retain intron 8, with SV4 also lacking exon 3. The remaining three splice variants lack exon 3 and retain intron 5 and its associated stop codon. SV5 and SV6 retain intron/s after intron 5, but the three SVs 5-7 encode the same protein sequence. Together the seven SVs encode five predicted proteins. Although five of the SV sequences contain stop codons in introns 5 or 8, the transcript continues through the remaining coding sections found in a typical *SERK *gene. In these sequences a second possible transcript occurs with a predicted start codon in exon 9 in the region encoding subdomain IV of the the kinase domain. This sequence continues through to the position of the stop codon in exon 11 of SV1 (usual SERK gene structure). This was confirmed by sequencing in SVs 4, 5, 6 and 7. In SV2, sequence data was not obtained for sequence corresponding to most of exon 10 and exon 11.

Although the *MtSERK3 *gene contains the typical 11 exon *SERK *genomic structure and SV1 has characteristics of a typical *SERK *transcript, there are some features that distinguish this gene from other *SERKs*. The first feature is the absence of a predicted signal peptide and the second is a truncated C-terminal domain, with the coding sequence terminating just after the kinase domain (Figure 1).

### Expression of Medicago *SERKs *during the induction of somatic embryogenesis in culture

**Figure 5:**
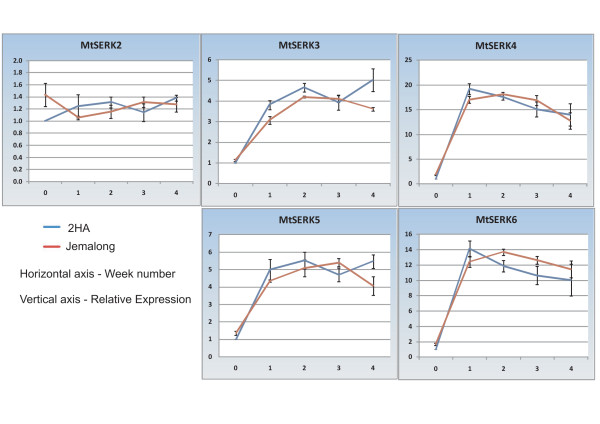
**Quantitiative RT-PCR (qPCR) expression studies of *MtSERKs 2, 3*,*4*, *5 *and *6 *in 2HA and Jemalong leaf tissue cultures over a four week culture period**. Results shown are means ± standard error of 3 biological repeats, calibrated to expression in the starting leaf tissue (week 0).

The apparent recent duplications of an ancestral gene to create the five *SERK *genes on chromosome 2 raised the question of whether or not the five Medicago genes are redundant in function of whether they have developed divergent functions. Our previous work showed that *MtSERK1 *expression is induced in somatic embryo-forming and root forming cultures [9] and we were interested to know if other *SERK *genes played a role in SE. Quantitiative RT-PCR (qPCR) expression studies were conducted on these five *MtSERKs *in cultured *M. truncatula *tissue. Relative expression was compared over a four-week time course in cultured leaf tissue from both the embryogenic 2HA seedline and the non-embryogenic Jemalong seedline (Figure 5). The expression of *MtSERK3 *was measured using primers that would amplify all putative splice variants of this gene. Therefore expression shown is the sum expression of all splice variants. Like *MtSERK1*, *MtSERKs 3-6 *are upregulated within the first week of culture and show similar expression in both the embryogenic 2HA and non-embryogenic Jemalong genotypes. These results show that *MtSERK1 *is not the only *SERK *gene induced in culture at the time of induction of SE. *MtSERKs 3 *and *5 *are upregulated four to five-fold over expression in the starting leaf material and remain relatively high over the four weeks. This is a similar expression pattern to that observed for *MtSERK1 *[9]. However, as the expression results for *MtSERK3 *do not distinguish between splice variants, it is not known which or how many splice variants contribute to these expression levels. Expression of *MtSERK4 *and *6 *are more significantly upregulated (12-20 fold) within the first week of culture, then the expression decreases slightly (but not significantly) over the culture time measured. The variation in expression pattern between *MtSERK2 *and the other replicated *SERK *genes indicate some differences in function.

## Discussion

### *SERK *genes identified in *M. truncatula*

Previous Southern analysis indicated there are probably five *SERK *genes in *M. truncatula *[9], but we have now identified a total of eight *SERK *or *SERKL *genes in addition to the previously characterised *MtSERK1*. Each of these nine genes contains 11 exons which is characteristic of *SERK *genes, as well as the tendency for each exon to encode a specific protein domain. Phylogenetic analysis shows that five of these genes are *SERKs*, belonging to the SERK 3/4/5 subfamily. The other three do not fall into the *SERK *family as defined in Arabidopsis, but rather are *SERK*-like genes. Two of them, *MtSERKL1 *and *MtSERKL2 *fall into the *NIK *family, which is highly similar to the *SERK *family. The third one, *MtSERKL3 *is also closely related but is not in the same clade as the *SERK *or *NIK *genes.

The carrot SERK does not contain a signal peptide, but rather starts from the leucine zipper (exon 2 in other SERKs). A perfect leucine zipper (Leu-X_6_-Leu-X_6_-Leu-X_6_-Leu [37]), is not present in AtSERKs 4 and 5 and the specific SPP motif of the SPP domain is also lacking in these sequences (Figure 1). However, phylogenetic analysis favours the view that these are still SERKs [40](Figure 3). The Arabidopsis *NIK *genes share many similarities with *SERK *genes. Several genes from other species that have been named as *SERK *genes fall in the same clade as the *NIK *genes (Figure 3). Function has not been identified for the three Arabidopsis genes that fall into the clade with MtSERKL3.

### SERK genes in legumes

Although the *M. truncatula *genome is not yet fully sequenced, we have attempted to identify all *SERK *genes in this species. From the identified *SERKs*, only one belongs to the SERK 1/2 subfamily (as defined in Arabidopsis), while there are five in the SERK 3/4/5 subfamily. This indicates there are probably not direct orthologues to the five Arabidopsis SERKs. Recently soybean became the first legume genome to be completely sequenced [41]. The soybean genome has 20 pairs of chromosomes and is a tetraploid, whereas the diploid *M. truncatula *genome has 8 pairs of chromosomes. It is estimated that the soybean genome underwent duplication around 13 million years ago and that any given region in the *M. truncatula *genome is likely to correspond to two regions in the soybean genome [42]. A search for candidate *SERK *and *SERK*-like known and predicted genes in soybean revealed 17 genes. Phylogenetic analysis showed that three of these fall into the SERK1/2 subfamily, in comparison to one in *M. truncatula*. Like Medicago, there are five putative SERK 3/4/5 subfamily members in soybean. Five members fall into the NIK clade and four are part of the clade, containing MtSERKL3, separate to SERK and NIK.

In evolutionary terms, the closest legume to *M. truncatula *that has *SERK *sequence information is Lotus. The divergence of Medicago and Lotus is estimated to have occurred around 50 million years ago, after the divergence of soybean from Medicago and Lotus around 54 million years ago [38]. The predicted gene in Lotus which appears to be orthologous to the five SERK3/4/5 family member genes is a single copy gene, indicating that the Medicago genes may have duplicated after the divergence of Medicago and Lotus. We estimate the duplication of the Medicago genes occurred much more recently - from 3.25 to 2.2 million years ago. Phylogenetically there are two soybean genes that are equally closely related to these five Medicago SERKs (Gm08g19270 (Gm2) and Gm15g05730; Figure 3). These genes occur on different chromosomes and would originate from duplication of the entire soybean genome rather that duplication of a single gene. However, duplication has occurred on a less closely related soybean SERK3/4/5 gene, with two genes occurring in tandem on chromosome 5 (Gm05g24770 and Gm05g24790; Figure 3). It appears that soybean had its own SERK3/4/5 family member duplication event after its divergence from Medicago and Lotus.

In the *SERK *and *SERKL *genes there is not a simple ratio of two soybean genes for every Medicago gene, as would be expected from simple duplication of the soybean genome. It may be that not all of the Medicago genes have been identified, especially those that are not in the SERK clade. On the other hand, there is the likelihood of genome changes in both of the species during the past 50 million years to produce the gene compliment that is identified. Full sequencing of the *M. truncatula *genome would be the only way to fully and conclusively elucidate the complement of these genes in *M. truncatula*.

### *SERK *and *SERKL *genes in relation to development and defence

We propose the similarities between *SERK *and *NIK *genes in both structure and function indicate that these gene families, as well as other closely related LRR-RLKs, form part of a larger gene superfamily that operates in signalling during plant development and defence. The families cannot be segregated based on developmental or defence function, with both families containing members in each type of role and some individual members operating in both pathways. For example, Os5 (Figure 3, SERK1/2 sub-family) has a dual role in somatic embryogenesis and defence against fungal pathogens [30], Os3 (Figure 3, SERK1/2 sub-family) is linked to fungal defense [43], so-called *PpSERK1 *and *PpSERK2 *(Figure 3, *NIK *family), act in the early defining stages of apomixis [44]. Therefore it may be advantageous to consider the wider SERK/NIK gene superfamily, encompassing all LRRII subclass genes, when looking at *SERK *gene function in plants.

### Expression of Medicago *SERKs *during the induction of somatic embryogenesis in culture

Historically legumes have been difficult to transform and regenerate. The model legume, *M. truncatula *can be transformed and regenerated via somatic embryogenesis, but there is first a requirement for selection of an amenable genotype using tissue culture [45-48]. A role for MtSERK1 during the establishment of embryogenic and organogenic cultures in *M. truncatula *was implied when it was shown that this gene is upregulated under these culture conditions. Expression of *MtSERKs 3, 4, 5 *and *6 *, like *MtSERK1 *is significantly upregulated in both 2HA and Jemalong cultured tissue in comparison to expression in the starting leaf tissue (week 0), whereas the expression of *MtSERK2 *remains constant throughout culture and in the starting leaf tissue. This variance in expression pattern suggests that MtSERK2 at least, functions differently to MtSERKs 3-6. *MtSERK4 *and *MtSERK6 *are the most highly upregulated *SERKs *in culture with both showing greater than 12 fold upregulation of expression in the first week of culture (Figure 5). The expression of the three Medicago *SERKL *genes stayed fairly constant in leaf tissue and in culture suggesting these genes are not part of the regulation of events in culture (data not shown).

Our intron analysis indicates that the *MtSERK2 *and *MtSERK3 *genes arose from the first duplication event, esimated to be 3.25 mya. This raises the possibility that any function dependent on upregulation of *MtSERKs 3 - 6 *in culture evolved after the first duplication event which may be of significance when comparing the embryogenic capacity of different legume species. It was also noted that the promoter sequences of the five replicated *M. truncatula SERK 3/4/5 *subfamily member genes show greater sequence divergence between the members than the intron sequences (data not shown). Such a rapid change in gene promoters also supports the theory of functional divergence of these genes. In *M. truncatula SERK1 *expression is associated with developmental change [35]. It seems likely that as in Arabidopsis, heterodimers involving SERK1 with other SERKs or other RLKs help to regulate legume development.

### Splice variant

To our knowledge, the detection of sequences consistent with the existence of splice variants of *MtSERK3 *is a novel observation for a *SERK *gene. An understanding of AS in plants is in its early days, but it is estimated that AS occurs in about 20% of plant genes. What is known is the predominant form of AS in plants is in the form of intron retention comprising around 50% - 60% of AS events, with exon skipping comprising around 8%. This is quite different from the situation in humans where exon skipping is the predominant form of AS (58%) and intron retention comprises around 5% [49,50]. Using sequence data from ESTs an attempt has been made to identify alternatively spliced genes in Arabidopsis, rice and legumes and this information has been deposited on the ASIP (Alternative Splicing in Plants) database at http://www.plantgdb.org/ASIP/[49,50]. As *MtSERK3 *is one of the genes that had no corresponding ESTs on the database, it is not listed in the *M. truncatula *splice variants on the ASIP database. None of the five Arabidopsis *SERK *genes are listed as having splice variants. However, AS events are recorded in other LRR-RLKs, and in a separate study 34 alternatively spliced LRR-RLKs were identified in Arabidopsis [51]. AS producing premature stop codons, such as SVs 2,4,5,6 and 7 of *MtSERK3*, may produce transcripts that are targets for non-sense mediated RNA decay [49]. However, Ner-Gaon (2004) [52] presented evidence that transcripts with retained introns are exported from the nucleus and are associated with ribosomal complexes thus supporting the view that they may be functional.

The seven splice variant sequences observed in *MtSERK3 *are predicted to code for five different proteins (Figure 4), including SV1 that has a regular SERK-like structure. The structure of SV2 and SV4 gives these proteins structural similarity to the known RLPs such as CLAVATA2 (CLV2) [53]. The SVs 5, 6 and 7, encode a single severely truncated predicted protein which contains the N-terminal, lacking LRR1, with a stop codon introduced immediately after the position of LRR4 in a normal SERK gene. This leaves the first two exons, encoding the putative SP and ZIP domains, then three LRRs followed by a stop codon. We have no knowledge of reports of a similar truncated LRR-RLK in the literature and it is quite conceivable that such a protein is targeted for degradation. On the other hand there are other defence proteins that are encoded by alternatively spliced genes where it has been shown that AS of these genes is necessary to enable the defence function [54]. For example, the Arabidopsis *RESISTANCE TO PSEUDOMONAS SYRINGAE4 *(*RPS4*) gene belonging to the Toll/interleukin-1 receptor (TIR)-nucleotide binding site (NBS)-Leu-rich repeat (LRR) class of disease resistance (*R*) genes is alternatively spliced to give both full-length and truncated proteins, and the presence of all of these proteins is required for disease resistance [55]. In general there is a bias for alternatively spliced genes with intron retention in plants to function in defence and external/internal stimuli-related functions [52]. Additionally, in the mouse, members of the TOLL-like receptor signalling pathway show widespread alternative splicing, which is thought to allow a higher level of diversity in the inflammatory pathway in response to pathogens [56]. The already established role for *SERK *genes in defence raises the possibility that some defence effect related to AS could be operating in *MtSERK3*. Of course such a role would need to be shown experimentally.

In keeping with a potential defence role, a recent study suggests that plant LRR-RLK genes can be grouped according to whether or not they have undergone gene expansion [57]. The authors propose that the expanded group share similarities with the NBS-LRR resistance genes in their genetic variation and evolution and are more likely to function in disease resistance, whereas the non-expanded group have a tendency to function in growth and development. The expansion of the five Medicago SERK3/4/5 family member genes from a single ancestor may imply a role in defence for one or more of them. This observation along with the similar gene splicing to that observed in TIR-NBS-LRR genes is supportive of a role for MtSERK3 in defence. The rice SERK1/2 family member gene, *OsBISERK1*, [43] (Os3 in Figure 3) is one example of a defence related gene belonging to the expanded group [57].

MtSERK3 has other unusual features. One is its lack of a predicted signal peptide, although it is predicted to be secreted in a non-classical manner. The other is the truncation of the C-terminal domain in comparison to other SERK proteins. The actual function of the SERK C-terminal domain is unknown, but one possibility is a role in protein-protein interactions [7]. The distinct characteristics of MtSERK3 may indicate a rapid evolution of different function after the gene duplication events which had created five genes from a single ancestral gene. The creation of extra gene copies relaxed selective pressure allowing some copies to evolve new functions, while at least one of the genes maintained the original function. However *MtSERK3 *is still upregulated in tissue culture which also implies a developmental role during early culture events similar to that of *MtSERK1 *and to other SERKs that were first described in relation to SE.

## Conclusions

In this study we have identified and sequenced the mRNAs of five more *SERK *and three *SERK-like *genes in *M. truncatula*, and used these sequences to identify homologous genes in soybean. Phylogenetic analysis shows that some of these genes fall distinctly in the *SERK *family, while others are *SERK-like *which include *NIK *genes and other LRRII subgroup RLK-LRR family members. The *M. truncatula *SERK3/4/5 subfamily genes have undergone a gene duplication event that is not present in orthologous genes in soybean or Lotus. One of these duplicated genes apparently encodes a number of sequences, consistent with the existence of splice variants, which is a novel finding for a *SERK *gene. The gene duplication event and the presence of splice variants may be indicative of a role in defence, similar to that observed in NBS-LRR genes. Other members of this replicated SERK3/4/5 gene cluster are upregulated in embryogenic tissue cultures implying a similar developmental role to that previously observed for MtSERK1 [9,35].

## Methods

### Degenerate PCR and database mining

Degenerate primers for PCR were designed in the conserved kinase domain of *SERK *genes. To give greater specificity at the 3' end of the primers, each primer was made into two separate primers with a specific nucleotide at a point of degeneracy close to the 3' end where there was a choice of 2 nucleotides (underlined bases in primer sequences below). This gave the forward primers 8 specific bases and the reverse primer 5 specific bases at the 3' ends. The primers used were Forward 1 - 5'- CAR TTY CAR CAN GAR GTN GAA ATG AT-3,' Forward 2 - 5'- CAR TTY CAR CAN GAR GTN GAG ATG AT-3', Reverse 1 5'- CC RTA NCC RAA NAC RTC NGT YTT TTC -3', Reverse 2 - 5'- CC RTA NCC RAA NAC RTC NGT YTT CTC -3.' The degeneracy was 256-fold for the forward primers and 1024-fold for the reverse primers, with a predicted amplicon size of around 446 bp. Degenerate PCR was performed on cDNA and genomic DNA using a 2 μM concentration of each primer. PCR cycling conditions were a denaturation step of 3 min at 95°C, 40 cycles of 95°C for 30 s, 52°C for 30 s and 72°C for 60 s, and then 1 cycle of 72°C for 7 min. PCR products were cloned into pGEM Easy vector (Promega) and sequenced. Sequencing of four cDNA clones revealed they all belonged to the same gene which corresponded to TC100619. Sequencing of 14 genomic clones gave four individual sequences, however one of them did not appear to be a *SERK *sequence. Genes that were detected using degenerate PCR are indicated in Table 1.

To conduct database mining the mRNA sequences of known *SERK *genes were blasted against *M. truncatula *sequences in the DFCI Medicago Gene Index and NCBI nr and htgs databases. Two genomic DNA regions containing *SERK*-like sequences and seven TCs or ESTs were identified in addition to the already annotated *MtSERK1*. The genomic DNA regions identified were on chromosomes 2 and 5, with the region on chromosome 2 containing multiple predicted *SERK*-like genes. Four of the detected TCs matched chromosome 2. The remaining three TC/ESTs matched regions on chromosomes 3, 5 and 8. A summary of all sequences is shown in Table 1. All of the *SERK *and *SERK-like *sequences identified using degenerate PCR corresponded to sequences identified from the database searches or matched *MtSERK1*.

The chromosomal location of each *M. truncatula SERK *and *SERKL *gene was determined by performing a genomic sequence CViT blast of each gene against the *M. truncatula *pseudomolecule: MT3.0 database http://www.medicago.org/genome/cvit_blast.php. The actual gene sequences were obtained from the Medicago GBrowse v3 database http://gbrowse.jcvi.org/cgi-bin/gbrowse/medicago/ by following the links from the CViT blast results, selecting the appropriate postion on the chromosome and downloading the sequence data. These were then compared with the sequence data for each gene obtained from NCBI and from sequencing. Some manual adjustment was required to locate the total gene sequence from some blast results. The corresponding gene loci numbers of each sequence were also obtained from the Medicago GBrowse v3 database. Matching probeset IDs were obtained by blasting mRNA sequences against the Mt Affy Chip Consensus Sequences database on the *M. truncatula *Gene Expression Atlas website http://mtgea.noble.org/v2/.

### PCR amplification and sequencing of full length coding regions

As none of the TC/EST sequences identified represented full length coding sequences, potential coding regions from the individual genomic sequences were predicted using FGENESH software (Softberry; http://linux1.softberry.com) and this information was matched with sequences obtained from the DFCI Medicago Gene Index. Primers were designed from the known and predicted coding sequences and in predicted 5' and 3' untranslated regions, and these were used to amplify full length or overlapping partial length cDNA sequences. These regions were sequenced, either directly from the purified PCR product or were cloned into pGEMeasy vector, electroporated into *E. coli *and sequenced after either miniprep or colony PCR. Using this system any previously unsequenced sections of mRNA transcripts were PCR amplified and sequenced, giving full length sequence data for all of the identified *M. truncatula *genes. The cDNA used to obtain the sequences came from different sources of plant tissue including, flower, leaf, root, seedling, cultured tissue and somatic embryos. Tissue from both 2HA and Jemalong seedlines was used to make cDNA. Mostly pooled cDNA samples from various tissues were used as a template source. Where possible a full length coding sequence was amplified in a single PCR reaction and used for sequencing. The three genes which did not have any transcript sequences on the database (*MtSERKs 3, 4 *and *6*) required nested PCR reactions or shorter overlapping PCRs to obtain full length product for sequencing. In the case of *MtSERK3*, numerous nested PCR and cloning reactions from different tissue sources were required to identify the various splice variant sequences. However all of the nested PCR products used for sequencing of splice variants, with the exception of SV2, were full-length or almost full length sequences to ensure the sequence obtained did indeed come from a single transcript. In the case of SV2 the nested reverse primer was in exon 10. In all cases the products of the first PCR reaction, used as a source of template for nested PCR, were created using primers that amplified the full length coding sequence of the gene. Primers used for genes amplified in a single PCR reaction were: *MtSERK2 *- forward primer 5'-TCTCATCTTTTTGCTTCCATTC-3', reverse primer 5'-AAAGTGTTGGTTGCTTGTGTC-3'; *MtSERK5 *forward primer 5'-GAGAGAGAGGGTTTGTGTTTT-3', reverse primer 5'-AGAGGACGGATTGTGTATTG-3'; *MtSERKL1 *- forward primer 5'-CTCCTTTACCTTTACCACACTTC-3', reverse primer 5'-ATCTACAACAACCCCAAATAACA-3'; *MtSERKL2 *- forward primer 5'-GGTTTCTTCTGCTGCTCTTTCTC-3', reverse primer 5'-CAGAAAGCTCCATTGCTTCTAC-3' and *MtSERKL3 *- forward primer 5'-AATTAAAGGGTTGGTTCATTCTT-3', reverse primer 5'-TCCAATCTGGTATGGTCTGT-3'. *MtSERK4 *was amplified in two overlapping PCR reactions using the primers - forward primer 5'-GCAAAGAAAACAAACAAAAGCCATAC-3' with reverse primer 5'-CTGGTGACGGTGGAGAAAGTG-3' and forward primer 5'-GAGATGTCCCCAAGAGTGGTTC-3' with reverse primer 5'-TTTATCTCGTTCAGGCAGAGGA-3'. *MtSERK6 *was amplified using nested PCR reactions. Primers for the first PCR were forward primer 5'-TGGAGTTTGATAATGGGTTTCTTG-3' with reverse primer 5'-CAGGCAGAGGAAGAAGGATTGT-3'. Products from the first PCR were diluted 1 in 100 and amplified using the same reverse primer and a nested forward primer 5'-TTTGGTTCTTCATTTGCTGCTTC-3'. Splice variant sequences for *MtSERK3 *were obtained using a number of nested PCR reactions followed by cloning and sequencing. For each splice variant the full length coding sequence was amplified in the first PCR reaction. The primers used for the nested PCR amplified a full length or almost full length coding sequence (except for SV2; sequence up to exon 10 obtained). A summary of the tissue and primers used in the PCR reactions to obtain the full SV sequences are given in Additional file 2.

### Gene and motif prediction

The genomic structure of the genes sequenced was determined using Spidey on the NCBI database http://www.ncbi.nlm.nih.gov/IEB/Research/Ostell/Spidey/. Prediction of mRNA sequences from genomic sequences was done using FGENESH software from Softberry http://linux1.softberry.com/berry.phtml. Predicted amino acid sequences from the sequenced genes were used for motif prediction using the ExPASy Proteomics tools server http://au.expasy.org/tools/. A general scan of the sequence was performed using Scan PROCITE [58]. Signal peptides were predicted using SignalP 3.0 [59]. Prediction of whether proteins could be secreted in a non-classical manner (without a signal peptide) was performed using Secretome 2.0 [60]. Due to the lack of plant parameters with this program, the protein was classed as mammalian for prediction. Transmembrane domains were predicted using TMHMM 2.0 [61]. MW and pI were estimated using ProtParam [62].

### Amino acid sequence alignments and phylogenetic analysis

Full length predicted amino acid sequences were aligned using ClustalX 2.0.10 [63]. Phylogenetic analysis was performed on aligned sequences using the protein maximum likelihood, proml, programme and tree topology edited using Retree from the PHYLIP (Phylogeny Inference Package) Version 3.69 http://evolution.genetics.washington.edu/phylip.html. Trees were drawn using TreeView 1.6.6 http://www.treeview.net/.

### Identification of putative SERK genes in soybean

A blast search of Gm genome soybean chromosomes (JGI Glyma1) on the Plant GDB database http://www.plantgdb.org/GmGDB/ was conducted using the mRNA sequences of each of the *M. truncatula SERK *genes to find homologous sequences in the soybean genome. From these searches it was possible to obtain the locus number of each of the matching genes. These loci numbers were used to obtain the corresponding genome and predicted mRNA and AA sequences from the Phytozome database http://www.phytozome.net/, which contains the full sequence of the recently sequenced soybean genome.

### Estimation of gene duplication events in *MtSERK *family members

To estimate the time of duplication of the MtSERK 3/4/5 subfamily member genes, each intron sequence of each gene was compared to the corresponding intron sequence in the other duplicated genes using the Needleman-Wunsch Global Sequence Alignment Tool at NCBI [64]. The following parameters were used for comparison: match cost 2, mismatch "-3", gap cost 5, gap extension 2. In some cases manual adjustment was necessary. The number of substitutions and deletions were counted and the age of duplication (distance) was calculated for each pair, using the assumption of 3 × 10^-10 ^substitutions/site/year [65], and also taking into account the fact that mutations occur independently in each copy after a duplication event. Comparison of the differences between different pairs of genes allowed the calculation of the sequence and approximate times of duplication events.

### Quantitative RT-PCR

*M. truncatula *2HA and Jemalong leaf tissue was collected, sterilised and cultured as described in [9]. RNA was isolated from 2HA and Jemalong cultured and leaf tissue using the RNAqueous-4PCR kit (Ambion) according to the manufacturer's instructions. All RNA samples were treated with DNase prior to cDNA synthesis. cDNA synthesis was performed using the SuperScript II first-strand synthesis system for RT-PCR (Invitrogen) from 2 μg of total RNA using oligo(dT) primers. All qPCR reactions were set up using the CAS1200 robot (Qiagen formerly Corbett) and run on the Rotor-Gene Q (Qiagen formerly Corbett). Primers were designed using Primer3 programme (Primer3 site http://frodo.wi.mit.edu/primer3/). Due to the similarity in sequence between the *M. truncatula SERK *genes, each primer was checked for specificity against an alignment of the other *M. truncatula SERK *genes. The amplified PCR products were tested for the presence of a single PCR product using a high resolution disassociation curve with temperature increasing in 0.2°C increments at the end of each PCR run. For some of the genes a number of different primer sets and annealing temperatures were tested to find conditions with specificity. Primer sequences used were: *MtSERK2 *- forward primer 5'- AGTTGAAGAAAAATGGAACAAGTGA-3', reverse primer 5'- TCAGTGCATCACCTTCAACATTAG-3'; *MtSERK3 *- forward primer 5'- GTGTATCGTGTTTACGAGAACGTAATGG-3', reverse primer 5-TCACGGTGAATAATCTTAGGGTCACA-3; *MtSERK4 *- forward primer 5'- CAATGAAGAAAGTGATGCCCTGAA-3', reverse primer 5'- CATCATTGCATCCAACATGAAACC-3'; *MtSERK5 *- forward primer 5'- CTTCTTCCAATGATGAAAGTGATGC-3', reverse primer 5'-ATCAACCCGGATTACTCTACCACCAC-3' and *MtSERK6 *forward primer 5'- CATCACCAGCTTCTTCAGGTAGCA-3', reverse primer 5'- GCAGGAACGTCAAAGAAATGATCC-3'. cDNA was diluted to 1 in 25 for qPCR reactions. Reactions were performed in triplicate in 15 μL sample volume using 0.3 units Platinum Taq PCR polymerase (Invitrogen), 1 × Platinum Taq reaction buffer, 3 mM CaCl_2_, 0.2 mM each of dATP, dCTP, dGTP, dTTP and 2 μM SYTO9 fluorescent dye (Invitrogen). PCR cycling conditions were 94°C for 2 min, followed by 40 cycles of 94°C for 15 s, 60°C for 30 s and 72°C for 30 s. For *MtSERK3 *and *MtSERK6 *PCR reactions the annealing temperature was increased to 64°C to increase the specificity of the primers. Gene expression was normalised to expression of *GAPDH*. *GAPDH *primers used were forward primer 5'- GACTTTATTGGTGATACCAGGTCG-3 and reverse primer 5'- GGTCAACCACACGGGTACTGTAA-3'. PCR efficiency of each run was calculated using the LinRegPCR programme http://LinRegPCR.nl[66]. Relative expression was calculated according to the method of Pfaffl [67]. Results shown are means ± SE of three biological repeats.

## Authors' contributions

KN conducted the experimental work, database mining, phylogenetic analysis and drafted the manuscript. SK compared Lotus and Medicago sequences and did the analysis of gene duplication events. RR supervised the analysis, discussed the results and critically revised the manuscript. All authors have read and approved the final manuscript.

## Supplementary Material

Additional file 1 **Sequence identity of mRNA sequences (top) and identity and similarity of amino acid sequences (bottom) of the *M. truncatula *SERK and SERKL with each other and with Arabidopsis SERKs and NIKs**. Tables are colour coded with darker colour indicating higher similarity. Click here for file

Additional file 2 **Summary of the nested or semi-nested PCR primers used to PCR amplify *MtSERK3 *splice variant mRNAs for sequencing, and the source tissue used as template for the first PCR reactions**. Click here for file
