# Supplementary material for: Characterisation of the legume SERK-NIK gene superfamily including splice variants: Implications for development and defence
Source: BMC Plant Biol. 2011 Mar 9;11:44. doi: 10.1186/1471-2229-11-44 (PMC3061892; doi:10.1186/1471-2229-11-44)
Supplement: Additional file 2 — Summary of the nested or semi-nested PCR primers used to PCR amplify MtSERK3 splice variant mRNAs for sequencing, and the source tissue used as template for the first PCR reactions. [file 1471-2229-11-44-S2.DOCX]

**Additional file 2**

Primers and tissue used to PCR amplify splice variants (SVs) of *MtSERK3* for sequencing

| SV number | Tissue | PCR1 forward primer | PCR1 reverse primer | Nested PCR forward primer | Nested PCR reverse primer |
| --- | --- | --- | --- | --- | --- |
| SV1 | Somatic embryos | For 1 | Rev 1 | For 4 | Rev 3 |
| SV1 | Pooled callus, flower and leaf | For 2 | Rev 2 | For 2 | Rev 4 |
| SV2 | Pooled callus flower leaf | For 2 | Rev 2 | For 2 | Rev 4 |
| SV3 | Flower | For 1 | Rev 3 | For 4 | Rev 2 |
| SV4 | Somatic embryos | For 1 | Rev 1 | For 4 | Rev 3 |
| SV4 | Root | For 3 | Rev 1 | For 3 | Rev 5 |
| SV5 | Somatic embryos | For 1 | Rev 1 | For 4 | Rev 3 |
| SV6 | Flower | For 1 | Rev 1 | For 4 | Rev 3 |
| SV7 | Somatic embryos | For 1 | Rev 1 | For 4 | Rev 3 |

**Primer sequences**

Forward primers

For 1 5’- TTGATGATCACAGTATCTTATGATGAGG - 3’

For 2 5’- AGGTGAGCCTGAACCCACTCTA - 3’

For 3 5’- TCCTTCTACAATCACTTTCAAGTAAATAA - 3’

For 4 5’- AGGTTGTCACAGGTGAGCCTGA - 3’

Reverse primers

Rev 1 5’- AACCTGATCAATGACCCCATGT - 3’

Rev 2 5’- AGAGTTCATCTGGCTCGATGTG - 3’

Rev 3 5’- CAGGCAGAGGAAGAAGGATTGT- 3’

Rev 4 5’- AGCGGAGGTTGAGAGCCATTAC - 3’

Rev 5 5’- TTCCTAGCTCTTCTGGGATTGTTCC - 3’
